# Germination rates of four Chilean forest trees seeds: *Quillaja saponaria, Prosopis chilensis*, *Vachellia caven*, and *Caesalpinia spinosa*

**DOI:** 10.12688/f1000research.16091.1

**Published:** 2018-09-10

**Authors:** Âlvaro Plaza, Miguel Castillo

**Affiliations:** 1 Universidad de Chile, Santiago, Chile

**Keywords:** germination; native forest; Mediterranean-climate zone

## Abstract

Data on the germination rates of four tree species, natively founded in the Chilean Mediterranean-climate zone, were determined by germination in crop chambers. The obtained data were used to interpolate or extrapolate the time taken for 50% of seeds to germinate in each case. These results are useful for regional native forest research and, in a broad sense, for its use in models to study germination dynamics in Mediterranean-climate zones.

## Introduction

Knowledge of the germination rates of a species means that future determination of this rate is unnecessary, preventing the waste of time and seeds.

*Quillaja saponaria* and *Vachellia caven* are two of the most representative trees in the Chilean Mediterranean forest ( Perez-Quezada & Bown, 2015), so information about these species will be useful for ecological investigation and restoration. *Prosopis chilensis* is vulnerable in the wild and is a key species of its community ( Valdivia & Romero, 2013); data about its propagation is important for conservation biologists.

In this article, we present the germination rates of seeds of *Q. saponaria*, *P. chilensis*, *V. caven*, and *Caesalpinia spinosa*. Dataset 1 contains the raw data from which these germination rates are calculated ( Plaza & Castillo, 2018).

## Methods

### Samples

All seeds were collected from adult trees. *Q. saponaria* seeds were collected in VIII Región, Chile; seeds from *V. caven*, *C. spinosa* and *P. chilensis* were from Región Metropolitana, Chile. The seeds were collected between February and April 2017. Information about collection was obtained from the seed provider, CESAF Antumapu, http://cesaf.forestaluchile.cl/.

**Figure 1.**
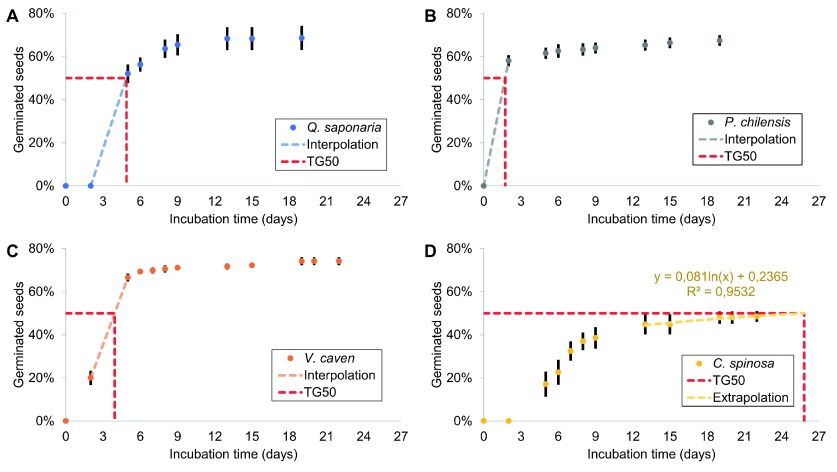
Time taken for 50% of seeds to germinate (TG50) for each species. Interpolation of *Q. saponaria* ( **A**), *P. chilensis* ( **B**) and *V. caven* TG50 ( **C**), and extrapolation of *C. spinosa* TG50 ( **D**).

Table 1 and Table 2 specify the initial number of seeds per plate and the percentage of germinated seeds in some days are shown. Figure 1 shows the obtained values of time taken for 50% of seeds to germinate (TG50).

**Table 1.** Percentage of germinated seeds of *Q. saponaria* and *P. chilensis* incubated for 19 days.

	Initial seeds per plate, n	Seeds germinated, %				
		Day 0	Day 2	Day 5	Day 13	Day 19
***Q. saponaria* (n=3 plates)**						
Average	**100**	**0.0**	**0.0**	**52.0**	**68.3**	**68.6**
Standard Error	10	0.0	0.0	4.3	5.4	5.6
***P. chilensis* (n=3 plates)**						
Average	**96**	**0.0**	**58.1**	**61.5**	**65.3**	**67.4**
Standard Error	4	0.0	2.6	2.6	2.5	2.4

**Table 2.** Percentage of germinated seeds of *V. caven* and *C. spinosa* incubated for 22 days.

	Initial seeds per plate, n	Seeds germinated, %						
		Day 0	Day 2	Day 5	Day 7	Day 13	Day 19	Day 22
***V. caven* (n=3 plates)**								
Average	56	0.0	20.0	66.6	69.9	71.7	74.2	74.2
Standard error	5	0.0	3.3	1.9	1.4	1.5	1.9	1.9
***C. spinosa* (n=3 plates)**								
Average	61	0.0	0.0	17.1	32.5	44.8	48.0	48.5
Standard error	3	0.0	0.0	5.8	4.5	4.7	2.9	2.5

### Pretreatment

Pretreatment conditions were suggested by the provider. Briefly, seeds of *Q. saponaria* were hydrated in tap water overnight. Seeds of *P. chilensis* were scarified in 95–97%, analytical grade H _2_SO _4_ for 10 minutes and then hydrated in tap water overnight. Seeds of *V. caven* were scarified in 95–97%, analytical grade H _2_SO _4_ for 90 minutes and then hydrated in tap water overnight. Seeds of *C. spinosa* were scarified in 95–97%, analytical grade H _2_SO _4_ for 30 minutes and then hydrated in tap water overnight.

### Germination

Activated seeds of *Q. saponaria*, *P. chilensis*, *V. caven*, and *C. spinosa* were placed in Petri plates over a filter paper bed (3 plates per species). Filter paper was then hydrated with distilled water. All plates were incubated in a crop chamber at 20°C, with light/dark cycles of 9 h/15 h. Germination is conditioned by temperature, so altering this factor could completely change the germination rates ( Giuliani *et al*., 2015).

Plates were monitored periodically to count the germinated seeds and refill distilled water. *Q. saponaria* and *P. chilensis* plates were monitored until day 19 ( Table 1). After that, fungal development made it difficult to check the plates, and a tactile examination of seeds indicated that most of them were rotten.

Plates containing *V. caven* and *C. spinosa* were more resistant to contamination and could be monitored until day 22. After this point, germination was too slow, and it was decided to end the experiment. Results are shown in Table 2.

The sample size, provided in the tables, is considered important for the replicability of a germination assay ( Ribeiro-Oliveira & Ranal, 2016).

### TG50 calculation

For *Q. saponaria*, *P. chilensis* and *V. caven*, the TG50 was linearly interpolated from the two closest points ( Figure 1A–C). *C. spinosa* didn’t reach the 50% germination during the assay, so this was extrapolated using the last five points ( Figure 1D). The TG50 of *Q. saponaria* was 4.9 days. *P. chilensis* had the fastest germination (TG50 = 1.7 days); *V. caven* had a TG50 of approximately 3.9 days, and the TG50 of *C. spinosa* was estimated to be 25.8 days.

Raw number of germinated seeds for each species, each repeat plate and each time point Also included are cumulative number of germinated seeds, percentages of germinated seeds and calculation of the TG50 for each species. Click here for additional data file. Copyright: © 2018 Plaza Â and Castillo M 2018 Data associated with the article are available under the terms of the Creative Commons Zero "No rights reserved" data waiver (CC0 1.0 Public domain dedication).

## Data availability

The data referenced by this article are under copyright with the following copyright statement: Copyright: © 2018 Plaza Â and Castillo M

Data associated with the article are available under the terms of the Creative Commons Zero "No rights reserved" data waiver (CC0 1.0 Public domain dedication). 

**Dataset 1. Raw number of germinated seeds for each species, each repeat plate and each time point.** Also included are cumulative number of germinated seeds, percentages of germinated seeds and calculation of the TG50 for each species. DOI: https://doi.org/10.5256/f1000research.16091.d216429 ( Plaza & Castillo, 2018).
