# Supplementary material for: Germination rates of four Chilean forest trees seeds: Quillaja saponaria, Prosopis chilensis, Vachellia caven, and Caesalpinia spinosa
Source: F1000Res. 2018 Sep 10;7:1446. [Version 1] doi: 10.12688/f1000research.16091.1 (PMC6259492; doi:10.12688/f1000research.16091.1)
Supplement: Peer review history

# Peer review: Referee response for version 1

**DOI:** 10.5256/f1000research.17571.r40998

**Published:** 2018-12-11

**Authors:** Diana Soriano

**Affiliations:** 1 Facultad de Ciencias, Universidad Nacional Autónoma de México, Mexico City, Mexico

Authors present germination data for four species from the Chilean Mediterranean forest. I would like to focus on the first statement of the introduction: 

*“Knowledge of the germination rates of a species means that future determination of this rate is unnecessary, preventing the waste of time and seeds”*.

 I think this statement is not accurate. Germination rate, especially in wild species, could be different depending on the cohort of seeds, the years of collection and the location. Data showed in this paper are valuable as a single biological replicate of germination behavior of the species used in the studies but it is necessary to add more biological replicates (different years and locations) to have a better understanding of germination behavior of the studied species.

 Methods:

1. I would like to know from how many trees seeds were collected.
2. TG50 calculation could be more easily reproduced if the authors fit their data to a model (p/e sigmoid) and calculated first maximum derivate.

I have read this submission. I believe that I have an appropriate level of expertise to confirm that it is of an acceptable scientific standard, however I have significant reservations, as outlined above.

# Peer review: Referee response for version 1

**DOI:** 10.5256/f1000research.17571.r39828

**Published:** 2018-11-23

**Authors:** Madelaine Quiroz Espinoza

**Affiliations:** 1 Faculty of Technology, University of Santiago, Chile (Usach), Santiago, Chile

The authors conducted germination trials on seeds of four Chilean forest tree species. They indicate that this information could be useful for conservation and restoration studies. Regarding the methodology, the description of pretreatments and germination trials stands out. The study design is appropriate and the work is technically sound. I believe that the authors could increase the number of replicates (Petri plate), but in this case it is correct as a first approximation to determine the germination rates of Chilean forest tree seeds.

 The manuscript represents a useful contribution to the theme of the germination of Chilean forest trees seeds and deserves to be indexed.

I have read this submission. I believe that I have an appropriate level of expertise to confirm that it is of an acceptable scientific standard.

# Peer review: Referee response for version 1

**DOI:** 10.5256/f1000research.17571.r39829

**Published:** 2018-11-08

**Authors:** Gabriela Saldías

**Affiliations:** 1 Facultad de Arquitectura, Urbanismo y Paisaje, Escuela de Arquitectura del Paisaje, Universidad Central de Chile, Santiago, Chile

The document provides valuable information on the germination rate of four native species. *Quillaja saponaria* and *Vachellia caven* are two of the most representative trees of the Chilean Mediterranean forest, *Prosopis chilensis* is in the category of threat and *Caesalpinia spinosa* adapts well to the conditions of the central zone of the country. The protocols for the collection of fruits and seeds, as well as the applied pre-germinative treatments, were based on methodologies recommended by the *Centro de Semillas de la Universidad de Chile, CESAF Antumapu*. Although they are described in the text, it would be convenient to add the references of Gold *et al.* (2004 ^1^) and INFOR (2015 ^2^) that complements the antecedents in pre-germination treatments for the species under study.

 With regard to the results obtained, knowing the time required to achieve 50% of seed germination is a useful fact that helps to plan the work of plant reproduction for research and ecological restoration purposes as proposed by the authors.

I have read this submission. I believe that I have an appropriate level of expertise to confirm that it is of an acceptable scientific standard.

## References

[1] GoldKLeón-LobosP: Manual de recolección de semillas de plantas silvestres para conservación a largo plazo y restauración ecológica. *Boletín INIA*.2004;110:62 p. Reference source

[2] INFOR: Propagación de especies forestales nativas de las zonas áridas y semiáridas de Chile. *INFOR, CORFO, INIA*.2015;47:135 p. Reference source
